# Stories and Communication Behaviors in Conflict

**DOI:** 10.3390/bs16010049

**Published:** 2025-12-25

**Authors:** Yiftach Ron

**Affiliations:** 1 School of Creative Arts Therapies, Faculty of Humanities and Social Sciences, Kibbutzim College, Tel Aviv 6250769, Israel; yiftach.ron@smkb.ac.il; 2 The Swiss Center for Conflict Research, Department of Communication, Faculty of Social Sciences, The Hebrew University of Jerusalem, Jerusalem 9190501, Israel

## 1. Introduction

Conflict-supporting narratives, memories and identities (Bar-Tal, 2020; Myshlovska, 2022; Yilmaz, 2021), as well as psychological factors—such as intergroup bias and self-serving bias at the personal level (Komlik, 2021), selective information processing, and resistance to change (Sun et al., 2019)—play a major role in the eruption and persistence of interpersonal disputes and intergroup conflicts (Böhm et al., 2020; Rovenpor et al., 2019). Studies of communication processes in situations of conflict demonstrate how attitudes, narratives and beliefs regarding the causes, the course and the resolution of the conflict, as well as perceptions regarding the self and the other, are shaped and expressed across various arenas of mass communication, social media and face-to-face communicative interactions (D. G. Ellis, 2019; Ron & Maoz, 2013; Zeitzoff, 2016). The works presented in the following sections of this article investigate and reflect on the communication strategies and practices employed by individuals and groups within these arenas in conflict situations. They also explore the interrelations among communication behaviors, stories, information, perceptions, conflict, and human relations.

At its essence, conflict stems from incompatible interests and goals (D. Ellis et al., 2016). While opposing interests between parties on the interpersonal or group level and competition over material resources and control are at the core of conflict, conflicts are made salient through the construction of identities, worldviews and narratives that play a destructive role in preserving the conflict, and in denying the legitimacy of the other. These opposing identities, perceptions and narratives associate the conflict with a heavy load of sentiments, including fear, disparagement, blame and grudge (Bar-Tal, 2020; Bar-Tal et al., 2009; Bliuc & Chidley, 2022; Jelić et al., 2021).

Conflicts may exist at the group or individual level. At the group level, conflict-supporting narratives, collective memories and ethos of conflict play a major role in the eruption and the persistence of intergroup conflicts, with the societies involved investing continuous efforts in the form of material and cultural resources to legitimize and maintain the dominance of these narratives (Golynchik, 2020; Grever & van der Vlies, 2017). In their work on socio-psychological barriers to conflict resolution, Halperin and Bar-Tal (2011) and Bar-Tal and Halperin (2013) note that an *ethos of conflict*, including group-based world-views and cognitive or motivational biases, prolongs intergroup conflicts. More specifically, the collective norms inherent in conflict zones—including ideological extremity, emotional negativity (Bar-Tal & Halperin, 2013), and the common transition from subtle, ambivalent intergroup bias toward more direct, aggressive, and blatant forms of discrimination and exclusion (Fiske, 2002)—are operationalized through media and leadership discourse. By communicating these norms to group members, such discourse not only fuels existing animosity (Hammack, 2011; Kalb & Saivetz, 2007) but also preserves and reinforces conflictive beliefs regarding the unchangeable nature of the conflict and the perceived malice of the outgroup (Cohen-Chen et al., 2014a, 2014b). Furthermore, it shapes ingroup perceptions characterized by the absolute justification and idealization of the collective self and the cultivation of a victimized collective identity (Zembylas & Bekerman, 2008), particularly within sectors and societies experiencing protracted intergroup conflicts (Cohrs et al., 2015; Hall, 2017; Ron et al., 2017).

Interpersonal conflicts often occur in the context of intimate relationships or organizational/workplace relationships (Hussain, 2020; Overall & McNulty, 2017), and may be caused by various factors, including differences in goals, values, beliefs and opinions; competition over power or limited resources; or miscommunication/gaps in sharing or interpreting information (Beheshtifar & Zare, 2013). However, the distinction between interpersonal and intergroup conflicts is often an artificial, theoretical construct that cannot always be maintained in reality. Because groups are inherently composed of individuals, there is a recursive interplay between interpersonal and intergroup conflict (D. Ellis et al., 2016). An example of this is the type of conflict known as ‘identity conflict’, which is rooted in intergroup distinctions (often ethno-political), but is manifested in interpersonal contexts and implicated in individual development and well-being (Taylor & Spencer, 2004).

The recursive relationship between the collective and the individual also manifests in conflict-related narratives and practices of communication. Narratives play a significant role at the personal and collective tiers: At the personal tier, narratives function as a framework for attributing meaning to ideological affiliations and lived experiences, and assist in the formation of identity, security, and favorable self-regard (Alber, 2017; Bamberg et al., 2011; De Fina, 2003, 2009; McLean et al., 2017, 2020; Park & Moon, 2022). At the collective tier, narratives serve as an organizational tool that strengthens social identity and promotes the establishment of a social consensus (Bekerman & Zembylas, 2009). Studies of narratives and group identities in situations of intergroup conflict emphasize their role in reinforcing the legitimacy of the group and of its actions (Bekerman & Zembylas, 2009; Gamaghelyan & Rumyantsev, 2021), in establishing the group identity, and in boosting the sense of security and social solidarity (Canetti et al., 2017; Klar & Baram, 2014; Uluğ et al., 2020). Conversely, narratives and group identities play a key role in preserving the conflict and even escalating it (Golynchik, 2020; Uluğ et al., 2020), in strengthening the exclusive and polarized identities that deny the legitimacy of the other (Hammack, 2009, 2011), and in shaping the emotions and beliefs that feed the conflict (Bar-Tal, 2020).

While considerable scholarship on intergroup conflict has investigated the causes and consequences of such conflicts, perspectives centered on communication have been predominantly interested in strategies and conditions for addressing and resolving them (Gjerazi, 2023). Communication processes play an important role in managing these conflicts, given that intergroup and particularly ethnopolitical conflicts are resistant to resolution due to factors such as identity, historical injustices, asymmetrical power relations, and cognitive biases, many of which are psychological and symbolic in nature (D. Ellis et al., 2016; Meyer et al., 2017). Communication processes are essential for both the expression and resolution of interpersonal disputes and intergroup conflicts. Moreover, emergent patterns of communication, such as social media, have fundamentally reshaped the landscape of these conflicts, offering novel channels for both contestation and resolution (D. G. Ellis, 2015).

Within this context, this Special Issue sought to bring together studies examining communication behaviors, strategies, practices, and structured interventions applied in conflict contexts, aiming to promote the reduction of prejudice and the improvement of interpersonal and intergroup relationships.

## 2. Communication Strategies and Practices in Conflicts

The studies included in this special issue offer different perspectives on the role of communication behaviors and practices, identity, story sharing and information processing in diverse contexts of conflict.

Two of the studies examine crisis communication, analyzing how controversial public health and political debates (specifically regarding the COVID-19 pandemic, vaccination, and healthcare policy) are shaped and amplified by user engagement on social media platforms. Atad and David (Contribution 1) examined the effect of one of three sources of information: a politician (authority figure), a physician (expert), and an ordinary person (non-expert) who appeared in a personal story related to a controversial issue (COVID-19 vaccination) on Facebook, on the willingness to engage with it. The study’s key findings show that engagement behavior is dictated by users’ prior trust or belief in conspiracy theories: respondents with high institutional trust preferred sharing a politician’s story, while “conspiracy believers” were more likely to comment on posts from an ordinary person. The study also revealed a higher likelihood of sharing the story in interpersonal conversations than in other communication types, regardless of the source. These findings contribute to our understanding of the mechanisms underlying the spread of stories and misinformation in the digital age and during times of crisis.

In a similar vein, Kumar et al. (Contribution 2) also address digital media communication practices in the aspects of health policy, and in the context of the COVID-19 pandemic. Their study analyzes the discourse surrounding Medicare-for-All (M4A) on Twitter by examining the Twitter feeds of two opposing health advocacy groups—Physicians for a National Health Program (PNHP) and Partnership for America’s Healthcare Future (P4AHCF) before and after the initial wave of the COVID-19 pandemic. The study results point to a difference between the two interest groups’ communication strategies. PNHP has higher engagement with Twitter users. It is more adaptive to a pandemic narrative and showed a consistent narrative before and after the onset of COVID-19. In contrast, P4AHCF showed more scientific terminology and data-centric tweets and had an inconsistent narrative with a sudden surge in positive sentiments and a complete silence on Medicare-For-All right after the initial wave of COVID-19. The findings add to our understanding of healthcare advocacy campaigns on social media and the implications of a pandemic for health policy reform.

Another study by Belgasm et al. (Contribution 3) presents a different perspective on communication behaviors in conflict situations. This study explores the complex interplay between interpersonal conflict, workplace ostracism, and interpersonal deviance in the public sector, emphasizing the moderating role of supervisors’ active empathic listening. The findings reveal that interpersonal conflict strongly predicts workplace ostracism and interpersonal deviance, while supervisors’ active empathic listening moderates these effects and reduces the likelihood of deviant behaviors. These findings underscore the importance of fostering empathetic leadership and inclusive workplace environments to mitigate conflict’s negative impact. This research contributes to understanding workplace dynamics by highlighting the critical role of supervisors in moderating conflict and ostracism.

## 3. Communication and Intergroup Relations in Intractable Conflict

Four of the articles in this Special Issue explore various aspects concerning intergroup relations in the context of intractable conflict, and more specifically, in the Israeli–Palestinian conflict.

A study by Rosler et al. (Contribution 4) explores the psychological mechanisms that underpin the effectiveness of the Informative Process Model (IPM) intervention, a novel tool designed to “unfreeze” deep-seated, conflict-supporting attitudes prevalent in societies involved in intractable conflicts. The research, using both quantitative and qualitative methods across two studies with Jewish-Israeli participants, specifically aimed to understand their experiences when exposed to IPM messages. The findings indicate the intervention’s effectiveness in facilitating attitude change by generating cognitive and emotional ambivalence towards existing conflict narratives. This ambivalence—the simultaneous holding of conflicting feelings or beliefs—serves as a critical mechanism that encourages individuals to disengage from their conflict-supporting narratives and become more open to contemplating new information, ultimately predicting increased support for peace negotiations.

Another study examining the potential of a novel tool for promoting intergroup communication and relations between Israelis and Palestinians is the research by Peled et al. (Contribution 5). This study introduces and evaluates the concept of Telerobotic Intergroup Contact, a novel approach designed to facilitate mutual, direct communication between members of groups involved in intractable conflict, specifically focusing on the Israeli–Palestinian context. The research uses two mixed-method studies—one with Israeli participants and one with Palestinian participants—to assess the acceptability, preferences, and anticipated utility of utilizing remote-controlled robots as mediators for intergroup contact. The findings indicate that, overall, participants show high levels of acceptance for using telerobots as a communication tool, viewing them as a potential method to overcome the barriers of physical distance, security risks, and the emotional difficulties associated with face-to-face interaction. However, the study also reveals differences in preferences, suggesting that the effectiveness of TIC may depend on the design and application tailored to each group’s unique needs and priorities in the conflict.

Within the same context, a different perspective is offered by Goldberg and Sliman (Contribution 6). Their study explores the effects of three different public and media representations of shared identity and tolerance on interreligious prejudice among Israeli Muslim adolescents and young adults. The interventions included an interfaith similarities-based common ingroup identity (focusing on shared aspects of Judaism and Islam), a modern national universalistic approach (focusing on religious tolerance), and a modern academic technological identity (highlighting Israel as a “Start-Up Nation”). Findings indicate that the interfaith similarities-based intervention had the most substantial impact in reducing prejudice, specifically by decreasing stereotypes and increasing willingness for intergroup contact. In contrast, the national universalistic and technological identity interventions were less effective in mitigating stereotypes and increasing willingness for social encounters. These findings point to the potential for leveraging interfaith commonalities as a foundation for intergroup prejudice reduction.

Lastly, Nasie’s study (Contribution 7) introduces and examines the concept of intergroup meta-respect, which refers to the belief that one’s ingroup is viewed by the outgroup as deserving of respect. The research, conducted among Jewish and Arab citizens of Israel, investigates perceptions of outgroup deservedness of respect, meta-respect, and their implications for intergroup attitudes. The study revealed systematic biases in meta-respect: Both groups, and to a greater extent the Jewish group, underestimated the extent to which the outgroup considered their ingroup deserving of respect as human beings. Exposure to corrective information increased participants’ feelings of respect, hope, and positive perceptions of the outgroup, and indirectly enhanced willingness to respect and interact with the outgroup. These findings highlight the potential held by interventions targeting respect perceptions for improving intergroup relations.

## 4. Conclusions

Taken together, the studies in this Special Issue investigate the role of communication, identity, story sharing and information processing in various conflict contexts, with a common focus on understanding behavioral dynamics and conflict mitigation. While contributions 1 and 2 address crisis communication and public health debates on social media, and contribution 3 shifts the lens to interpersonal conflict in the workplace, the remaining studies (Contributions 4–7) share a focus on intergroup relations within the context of the intractable Israeli–Palestinian conflict, exploring interventions ranging from attitude change and technology-supported contact to identity-based interventions aimed at prejudice reduction, and interventions targeting respect perceptions as a means for improving intergroup relations.

In addition to these studies, future research should aim to broaden the contextual scope. While this Special Issue provides rich insights into the Israeli–Palestinian context and public health crises, comparative studies are needed to test the effectiveness of models like IPM and meta-respect across other settings of conflicts. Furthermore, the domain of interpersonal conflict needs further exploration. There is room to investigate communication dynamics—such as empathetic active listening—in mitigating conflict outcomes within additional contexts. Moreover, given the emergence of generative AI and novel digital platforms, research must urgently examine the utility, acceptability, and ethical challenges of leveraging new technologies, including AI tools and automated communication technologies, for conflict resolution and improving both interpersonal and intergroup relations.
